# Variant‐to‐Biomarker Pathways in Peripheral Artery Disease: Multiomics Integration and Clinical Translation

**DOI:** 10.1155/humu/1908331

**Published:** 2026-04-29

**Authors:** Wanting Wang, Gang Zhao, Changxin Yang, Siyao Chang

**Affiliations:** ^1^ Heilongjiang University of Chinese Medicine, Harbin, China, hljucm.edu.cn; ^2^ Department of Peripheral Vascular Disease, The First Affiliated Hospital of Heilongjiang University of Chinese Medicine, Harbin, China, hljucm.edu.cn; ^3^ School of Medicine and Health, Harbin Institute of Technology, Harbin, China, hit.edu.cn; ^4^ Department of Vascular Surgery, Harbin Fifth Hospital, Harbin, China

**Keywords:** fine mapping, functional genomics, genome-wide association studies, multiomics integration, peripheral artery disease, variant-to-biomarker pathways

## Abstract

Peripheral artery disease (PAD) is a prevalent, disabling manifestation of systemic atherosclerosis that carries high risks of major adverse cardiovascular and limb events, yet remains incompletely explained by conventional risk factors and haemodynamic indices. Although genome‐wide association studies have nominated reproducible susceptibility loci and high‐throughput profiling has expanded the landscape of circulating, imaging, vascular and skeletal muscle biomarkers, most signals are noncoding, mechanistic attribution is often uncertain and few biomarkers have demonstrated durable incremental utility for risk stratification or therapeutic guidance in routine care. In this review, we summarise PAD‐relevant genetic architectures and multiomics modalities—fine‐mapped GWAS with tissue‐ and cell‐resolved functional genomics, proteogenomic and metabolomic profiling and network‐based integration across vascular, muscle and circulating compartments—and we appraise translational opportunities that span variant‐anchored protein and metabolite prioritisation, composite biomarker panels for limb‐specific ischaemic burden and residual atherothrombotic risk and biomarker‐informed selection of antithrombotic, lipid‐lowering, anti‐inflammatory and revascularisation strategies. We also discuss enduring challenges—including ancestry‐sensitive transferability of genetic instruments, limited access to disease‐relevant tissues, cross‐platform standardisation, confounding by disease stage and therapy and the need for prospective validation and trial‐ready pharmacodynamic endpoints—that temper implementation. The purpose of this review is to delineate variant‐to‐biomarker pathways in PAD and specify integrative, clinically actionable solutions for discovery, validation and translation. We further distinguish diagnostic, prognostic, predictive/theragnostic and pharmacodynamic biomarker contexts of use, and emphasise that phenotype definition, sex, diabetes, exposure measurement and treatment effects all condition the interpretation and transferability of PAD multiomic signals.

## 1. Introduction

Peripheral artery disease (PAD) is a chronic atherosclerotic disorder of the lower extremity arteries and a major cause of global disability and cardiovascular mortality [1, 2]. Estimates based on Global Burden of Disease data and population‐based cohorts indicate that the number of individuals living with PAD has risen from roughly 100 million in 1990 to more than 200 million in recent years, with approximately 230–240 million cases reported for 2019 [3]. The disease burden is unequally distributed, with faster growth of prevalence and disability in low‐ and middle‐income regions than in high‐income countries and a higher prevalence and disability burden among women despite greater mortality among men [4]. PAD severely impairs walking capacity, quality of life and work ability, and is associated with substantially elevated risks of myocardial infarction, stroke and cardiovascular death even in patients with mild or asymptomatic disease [5]. Conventional risk factors such as tobacco use, diabetes, hypertension, dyslipidaemia and ageing account for a large proportion, but not all, of the observed disease burden [6]. These observations underscore PAD as a systemic manifestation of atherosclerotic cardiovascular disease (ASCVD) with complex, incompletely understood pathobiology.

Several concrete clinical problems make this biomarker agenda relevant in PAD. First, PAD is frequently underdiagnosed because symptoms are atypical or absent and ABI testing is underused, whereas patients who do come to attention often represent biologically heterogeneous phenotypes despite similar haemodynamic severity [1, 5, 7]. Second, clinicians need better tools to distinguish systemic atherothrombotic risk from limb‐specific risk of walking decline, tissue loss, poor wound healing and amputation, because these outcomes are only partly captured by conventional risk factors and resting ABI [1, 5, 8]. Third, treatment selection remains imprecise: the same patient may be considered for intensified antithrombotic therapy, aggressive lipid lowering, supervised exercise, endovascular or surgical revascularisation or combinations thereof, but current decision‐making has limited molecular guidance [1, 8, 9]. Biomarker thinking can realistically help by enriching detection in high‐risk but underrecognised patients, refining prognosis for both major adverse cardiovascular events and major adverse limb events, identifying biological endotypes more likely to benefit from specific interventions and providing pharmacodynamic readouts of treatment response [8–10].

Sex differences further complicate interpretation of PAD biology. Women have at least comparable, and in some settings greater, prevalence and disability burden, yet they are more likely to present later, with atypical symptoms, poorer walking performance and faster mobility decline [4, 8]. Potential contributors include smaller vessel calibre, differences in arterial stiffness and microvascular reserve, menopause‐related loss of oestrogen‐mediated vasodilatory and anti‐inflammatory effects, sex‐specific patterns of body composition and skeletal muscle reserve and persistent disparities in recognition and referral. Accordingly, biomarker signals may differ by sex not only because of exposure burden, but also because hormonal and vascular biology influence endothelial function, collateral adaptation, inflammatory tone and the translation of a given stenosis into disability.

Although PAD shares many pathophysiological features with coronary and cerebrovascular atherosclerosis, several aspects are distinct. Lower limb arterial segments are exposed to unique haemodynamic and mechanical stresses, and the downstream skeletal muscle develops a characteristic ischemic myopathy with mitochondrial dysfunction, metabolic reprogramming and tissue remodelling [11]. Clinical manifestations range from asymptomatic disease to intermittent claudication and chronic limb‐threatening ischemia, and many patients present with atypical symptoms or remain undiagnosed until advanced stages [7]. Epidemiological and twin studies indicate a heritable component to PAD susceptibility, supporting a role for genetic determinants beyond acquired risk factors [12–14]. Large genome‐wide association studies (GWAS) have now identified multiple loci associated with PAD. In the Million Veteran Program, a trans‐ethnic GWAS of over 31,000 PAD cases and 200,000 controls discovered 19 risk loci, several shared with coronary artery disease and large‐artery stroke, implicating lipid metabolism, thrombosis and inflammation pathways [15, 16]. More recently, multitrait analyses that jointly model PAD, coronary disease, venous thromboembolism and other vascular phenotypes have revealed additional variants with pleiotropic effects, further supporting a shared, polyvascular genetic architecture [17, 18]. However, as in other complex cardiovascular disorders, these loci explain only a modest proportion of overall heritability and the majority of risk variants reside in noncoding regions, where they are presumed to act through cis‐regulatory elements and other mechanisms that influence gene expression in specific vascular and immune cell types [19]. Systematically connecting these variants to downstream molecular traits and clinically measurable biomarkers remains a central challenge.

Conceptually, PAD biology can be partitioned into a shared atherosclerotic core and a limb‐specific response. The shared component overlaps with coronary and cerebrovascular disease and includes apoB‐containing lipoproteins, chronic inflammation, thrombin generation, platelet activation, endothelial injury and smooth muscle cell (SMC) phenotypic modulation [8, 10, 15–19]. The limb‐specific component reflects lower extremity haemodynamics and tissue context: long diffuse femoropopliteal or infrapopliteal disease, impaired vasoreactivity, microvascular dysfunction, medial calcification, ischemic myopathy of the calf, altered exercise physiology and wound‐healing biology in the setting of diabetes or tissue loss [8, 11, 20–25]. Distinguishing these layers matters because a circulating marker may primarily report systemic plaque burden, local limb ischaemia or both.

Ischemic myopathy is a major limb‐specific feature of PAD. Repeated cycles of exercise‐induced demand and inadequate oxygen delivery expose calf muscle to chronic ischaemia‐reperfusion injury, promoting mitochondrial DNA damage, impaired oxidative phosphorylation, reduced fatty‐acid beta‐oxidation, myofiber atrophy, denervation‐like remodelling and excess extracellular matrix deposition [11, 20–24]. As a result, exercise can trigger exaggerated lactate accumulation, acylcarnitine overflow, altered amino‐acid flux and other metabolic shifts that are detectable in plasma or across the femoral arteriovenous bed even when resting haemodynamic indices appear similar [20, 21, 26–31]. These features help explain why metabolomic signatures may track walking impairment and limb symptoms more closely than static anatomical measures alone.

Parallel advances have transformed the biomarker field in PAD. Beyond established haemodynamic and imaging measures such as the ankle–brachial index, numerous circulating biomarkers reflecting inflammation, thrombosis, lipid metabolism and myocardial injury have been associated with PAD risk and prognosis, yet few have achieved robust incremental value over conventional risk scores in clinical practice [8]. High‐throughput metabolomics and lipidomics have identified PAD‐associated perturbations in amino acids, acylcarnitines, ceramides and cholesteryl esters, and suggest that metabolic signatures differ across disease stages and may capture skeletal muscle and vascular pathology not reflected by traditional measures [20, 21]. At the tissue level, single‐cell and spatial transcriptomic profiling of gastrocnemius muscle from patients with PAD has revealed extensive transcriptional remodelling of endothelial and immune cell populations, including altered ATF3/ATF4‐positive endothelial subsets and LYVE1‐high macrophages that appear to modulate angiogenesis, extracellular matrix organisation and inflammatory signalling during chronic limb ischemia [22]. These studies demonstrate that PAD is characterised by coordinated changes across multiple molecular layers in both vascular and skeletal muscle compartments. At the same time, they also highlight marked heterogeneity between individuals and datasets, and the difficulty of inferring causal mechanisms or clinically useful biomarkers from single‐omic studies alone.

Multiomics integration offers a principled route to link genetic variation, cellular programmes and biomarker candidates in PAD. Integrative frameworks developed in ASCVD more broadly combine genomics, epigenomics, transcriptomics, proteomics and metabolomics with clinical phenotypes to prioritise molecular traits that lie on causal pathways and improve risk stratification beyond traditional factors [10, 32]. In this context, variant‐to‐biomarker pathways can be conceptualised as chains in which inherited or acquired genomic variation perturbs regulatory elements, alters gene and protein networks within specific vascular or immune cell populations and generates circulating or imaging biomarkers that are measurable in patients and predictive of clinical outcomes. Establishing such pathways in PAD requires the coordinated use of fine‐mapped GWAS signals, functional genomics data from disease‐relevant tissues, systems‐level network modelling and robust longitudinal cohorts with deep molecular phenotyping. By focusing on PAD‐specific genetic loci, multiomics signatures across vascular and circulating compartments, and analytical strategies that connect them, this review is aimed at delineating variant‐to‐biomarker pathways that are mechanistically informative and clinically actionable for risk prediction and therapeutic guidance in patients with PAD.

## 2. Genetic Risk Variants Associated With PAD

Genetic studies over the past two decades have established that PAD is a moderately heritable disorder, with family and twin data suggesting that 20%–30% of interindividual variation in susceptibility is attributable to inherited factors beyond conventional risk exposures such as smoking, diabetes and dyslipidaemia [12–14, 33]. These observations have been complemented by early candidate‐gene investigations of thrombosis, inflammation and lipid pathways, although heterogeneous case definitions and small sample sizes limited reproducibility. Meta‐analytic synthesis of this literature indicates that, among > 200 investigated variants, only a small subset in genes such as intercellular adhesion molecule‐1, IL6, and hepatic lipase shows consistent association with PAD diagnosis, and even these signals have not always been recapitulated in genome‐wide analyses [34, 35]. Together, these findings support a polygenic architecture in which many common alleles of modest effect, rather than a few high‐impact coding variants, shape PAD risk.

The largest advances have come from multiancestry GWAS leveraging biobanks with electronic health record–derived PAD phenotypes. In the Million Veteran Program, ~32 million variants were tested in more than 31,000 PAD cases and 210,000 controls across European, African and Hispanic veterans, with replication in UK Biobank [36, 37]. This study identified 19 genome‐wide significant loci, 18 of which had not previously been linked to PAD, and demonstrated that 11 signals (including LDLR, LPL and LPA) are shared with coronary artery disease and large‐artery stroke, whereas several others appear specific to PAD and severe limb outcomes such as tissue loss and amputation. Systematic review of GWAS and candidate‐gene studies confirms that these 19 loci currently represent the most robustly replicated common‐variant associations for PAD, despite substantial variability in phenotyping and control selection across cohorts.

Phenotype definition is another major source of heterogeneity in PAD genetics. EHR‐derived PAD usually prioritises diagnostic codes, medication histories, procedure codes or narrative data and therefore enriches for clinically recognised and often more advanced disease, but it can misclassify asymptomatic PAD and may overrepresent patients who interact frequently with vascular care [15, 16, 33, 36, 37, 15]. ABI‐defined PAD anchors case definition to haemodynamic impairment and is attractive for epidemiology and early disease detection, yet ABI can be distorted by medial arterial calcification or noncompressible vessels, especially in diabetes and chronic kidney disease, and it incompletely captures exercise‐induced symptoms or distal perfusion failure [1, 5, 13]. Imaging‐defined PAD emphasises plaque burden, stenosis distribution, calcification, perfusion or collateral anatomy, whereas procedure‐based definitions preferentially capture patients selected for revascularisation or amputation and therefore reflect access to care, anatomy, symptom burden and clinician decision‐making in addition to biology [1, 8, 9, 36]. These distinctions matter because loci discovered in one phenotype may preferentially tag systemic atherosclerosis, calcific/noncompressible disease, limb‐threatening ischaemia or treatment‐seeking behaviour, and biomarker panels developed in one setting may not transfer directly to another without phenotype‐aware validation.

Functional annotation of PAD loci indicates that most risk variants reside in noncoding regions and are enriched in cis‐regulatory elements active in vascular endothelium, SMCs, hepatocytes and immune cells. Colocalisation with arterial expression quantitative trait loci (eQTL) and plasma protein quantitative trait loci has implicated genes involved in lipoprotein metabolism (LDLR, LPL, LPA and SORT1), coagulation and fibrinolysis (F5, F11 and PROCR), vascular matrix and remodeling (COL4A1/COL4A2), metabolic signalling (TCF7L2) and immune regulation (IL6R and SH2B3) as downstream mediators of GWAS signals [15, 33, 38]. Several PAD loci also overlap with variants associated with smoking behaviour (e.g., at the CHRNA3–CHRNA5–CHRNB4 cluster), underscoring the intertwined roles of behavioural and biological pathways in disease susceptibility [15, 33, 39]. These annotations support a model in which PAD risk variants exert their effects primarily through quantitative modulation of gene expression and circulating protein levels rather than disruption of coding sequence.

As shown in Table 1, risk loci identified to date can be organised into a limited number of biological categories that are directly linked to candidate biomarker axes. Loci at LDLR, APOB, PCSK9, LPL and LPA converge on lipoprotein metabolism and remnant lipoprotein clearance and are expected to influence plasma LDL cholesterol, triglyceride‐rich lipoproteins and lipoprotein(a). Coagulation‐related loci such as F5 and F11 highlight a thrombotic component that may correspond to variation in coagulation factor activity, D‐dimer levels and prothrombotic composite scores [40]. Variants near COL4A1/COL4A2, VEGFA and ANGPTL4 emphasise arterial wall structure, angiogenesis and extracellular matrix turnover, which may be reflected in imaging‐derived phenotypes including ankle–brachial index, plaque burden and microvascular perfusion measures [41]. Inflammatory and immune loci (e.g., IL6R and SH2B3) point towards biomarker signatures encompassing high‐sensitivity C‐reactive protein, IL‐6 family cytokines and activation markers in circulating leukocyte subsets [42]. Loci that primarily influence glycaemic control, adiposity or blood pressure act through established cardiometabolic intermediates but still contribute independently to PAD risk, suggesting that integrated biomarker panels will need to capture both traditional and genetically informed axes of risk.

**Table 1:** Major categories of common genetic risk loci for PAD and their putative biomarker readouts.

Category	Representative loci/genes	Principal biological process	Anticipated biomarker layer(s)
Lipid and lipoprotein metabolism	LDLR, APOB, PCSK9, LPL, LPA and SORT1	Regulation of LDL and remnant lipoproteins, triglyceride hydrolysis and lipoprotein(a) synthesis	Circulating LDL‐C, non‐HDL‐C, triglycerides, remnant cholesterol, apolipoprotein B, lipoprotein(a) and lipidomic profiles
Thrombosis and haemostasis	F5, F11, PROCR and FGG	Coagulation cascade activation, fibrin generation and protein C pathway	Coagulation factor activity assays, thrombin generation, D‐dimer and global thrombosis scores
Vascular structure and remodelling	COL4A1/COL4A2, VEGFA and ANGPTL4	Basement membrane integrity, angiogenesis and extracellular matrix turnover	Ankle–brachial index, arterial stiffness indices, imaging of plaque burden and neovessel density and matrix turnover peptides
Inflammation and immune signalling	IL6R, CXCR2, SH2B3 and HLA region	Cytokine signalling, leukocyte trafficking and adaptive immune activation	High‐sensitivity C‐reactive protein, IL‐6 family cytokines, leukocyte subset phenotypes and inflammatory proteomic signatures
Cardiometabolic traits and risk factor pathways	TCF7L2, GCKR, PPARG and blood pressure and adiposity loci	Glucose homeostasis, hepatic lipid handling, adipocyte biology and blood pressure regulation	Glycaemic indices, insulin resistance markers, body‐composition measures, blood pressure trajectories and metabolomic profiles

Beyond individual loci, several lines of evidence now indicate that PAD risk is driven by the cumulative effect of a large number of common variants with very small effect sizes. Genome‐wide polygenic risk scores constructed from PAD GWAS summary statistics, including those derived from the Million Veteran Program, capture additional heritable risk beyond the genome‐wide significant loci and show modest but consistent improvements in discrimination and reclassification when added to clinical models in external cohorts [33–36, 43]. Similar to polygenic scores for coronary artery disease, these PAD‐specific scores appear to be most informative in younger individuals and in those without substantial traditional risk factor burden, in whom they may highlight a subset with accelerated atherosclerotic susceptibility. However, performance remains sensitive to ancestry, the definition of PAD used for training, and the extent to which polygenic risk reflects pathways that are already captured by conventional biomarkers, such as lipids and blood pressure.

Despite these advances, current genetic discoveries explain only a fraction of PAD heritability, and the majority of associated variants have not yet been linked to specific cellular programmes or clinically measurable biomarkers. Integration of fine‐mapped GWAS signals with tissue‐ and cell type‐resolved epigenomic, transcriptomic, proteomic and metabolomic data, an approach that has already proven effective in other complex traits such as craniofacial malformations, will be essential to move from association to mechanism.

## 3. Multiomics Signatures of PAD Across Vascular and Circulating Compartments

### 3.1. Vascular and Skeletal Muscle Transcriptomic Landscapes

Multiomics profiling in PAD has increasingly focused on disease‐relevant primary tissues, particularly atherosclerotic arterial segments and ischemic limb muscle. Single‐cell RNA sequencing of human atherosclerotic plaques has revealed marked heterogeneity among SMC states, including a distinct SMC subset with high metabolic activity and broad differentiation potential that interacts extensively with macrophages and monocytes and shapes the inflammatory and metabolic microenvironment of advanced lesions [44, 45]. Although these data were generated largely in coronary and aortic plaques, the same cell types and regulatory programmes are implicated in femoral and tibial disease, supporting a shared multicellular atherosclerotic core that is modulated by vascular bed–specific haemodynamic and mechanical cues. In the distal arterial tree, RNA‐sequencing of peripheral vascular SMC isolated from tibial arteries of patients with critical limb ischaemia has identified differentially expressed genes linked to contractile–synthetic phenotypic switching, extracellular matrix remodelling and procalcific signalling under high‐phosphate conditions, including UNC5B and integrin‐related pathways, thereby connecting medial arterial calcification to distinct transcriptomic states in peripheral arteries [46, 47]. These tissue‐level data complement genetic findings by nominating specific vascular cell populations and gene networks through which PAD risk loci and cardiometabolic exposures may exert their effects.

In the limb musculature, scRNA‐seq and bulk transcriptomic analyses of chronic limb‐threatening ischaemia have shown that ischemic skeletal muscle is characterised by expansion of proinflammatory macrophage subsets, depletion and premature differentiation of satellite cells and reduced representation of endothelial and pericyte populations, together with broad induction of hypoxia‐responsive and stress‐response gene programmes [23, 24]. Pericyte‐focused RNA‐sequencing in experimental hindlimb ischaemia further demonstrates dynamic regulation of genes involved in angiogenesis, extracellular matrix organisation and myogenesis, with early upregulation of cytokine and cell‐cycle pathways and later enrichment of transcripts related to muscle fibre maturation and tissue repair [25]. These datasets delineate a multicellular muscle niche in PAD in which persistent ischaemia drives a shift towards proinflammatory myeloid states, impaired regenerative capacity, microvascular rarefaction and maladaptive matrix remodelling. As shown in Table 2, these tissue‐based transcriptomic and epigenetic studies establish a framework in which cell type–specific signatures from the arterial wall and skeletal muscle can be aligned with circulating proteomic and metabolomic profiles to define cross‐compartmental axes of disease activity.

**Table 2:** Multiomics data layers and vascular versus circulating compartments interrogated in peripheral artery disease.

Omics layer	Primary compartment and sample type	Representative molecular readouts	Dominant pathophysiological themes in PAD
Genomics/epigenomics	Blood‐derived DNA; arterial wall biopsies	GWAS loci, methylation marks and chromatin accessibility at vascular and immune enhancers	Polygenic risk, cis‐regulatory variation in SMC, endothelial and immune cells; links to lipids, thrombosis and inflammation
Transcriptomics (bulk and single‐cell)	Femoral/tibial arterial segments; skeletal muscle (gastrocnemius, tibialis anterior); vascular smooth muscle cells; pericytes	Cell type–specific gene expression, alternative splicing and pathway enrichment	SMC phenotypic modulation, medial calcification, endothelial dysfunction, proinflammatory macrophage states, satellite cell exhaustion and hypoxia‐response programmes
Proteomics	Plasma; platelet‐poor plasma; sometimes plaque tissue	Targeted and untargeted protein panels and pathway‐level protein modules	Complement and coagulation cascades, apolipoproteins and transport proteins, adhesion and signalling receptors, markers of endothelial activation and thromboinflammation
Metabolomics/lipidomics	Plasma or serum; occasionally urine	Amino acids, acylcarnitines, TCA intermediates, ceramides, sphingolipids and lipoprotein‐associated lipids	Mitochondrial dysfunction, impaired *β*‐oxidation, glycolytic shift, altered arginine/NO metabolism and lipid‐mediated vascular injury
Integrative and network analyses	Combined vascular, muscle and plasma datasets; external eQTL/pQTL/mQTL resources	Coexpression and coabundance networks, cross‐compartment modules, genetically anchored protein and metabolite signatures	Identification of molecular endotypes, mapping of variant‐to‐gene and gene‐to‐biomarker pathways, prioritisation of therapeutic targets and composite biomarker panels

### 3.2. Circulating Proteomic and Metabolomic Signatures

Proteomic profiling has yielded several classes of circulating biomarkers that mirror vascular wall and muscle pathology in PAD. Early candidate‐based and discovery proteomics in peripheral arterial disease reported associations of *β*_2_‐microglobulin, cystatin C, high‐sensitivity C‐reactive protein and other inflammatory and renal function markers with PAD presence and prognosis, and highlighted the potential of mass spectrometry–based plasma proteomics to refine risk stratification beyond the ankle–brachial index and traditional risk factors [48, 49]. More recent untargeted plasma proteomic studies in broader cardiovascular cohorts have identified coordinated changes in complement components, coagulation factors, acute‐phase proteins and lipoprotein‐associated proteins, implicating pathways such as IL‐6 signalling, LXR/RXR activation and endothelial activation, which are also central to PAD pathobiology [50, 51]. In PAD‐specific cohorts, systems‐level proteomic analyses of patients with high on‐treatment platelet reactivity to aspirin and clopidogrel have defined distinct protein signatures involving adhesion receptors (e.g., PSGL‐1 and PECAM1), thrombomodulin, protease‐activated receptor 1 and apoptosis‐related receptors, with several proteins showing graded associations with disease severity and antiplatelet response [52, 53]. These findings suggest that proteomic panels capturing thromboinflammatory activity may serve both as mechanistic readouts of plaque and platelet biology and as candidate biomarkers for treatment selection.

From a mechanistic perspective, limb events in PAD are tightly linked to platelet and coagulation biology. Atherosclerotic plaque disruption or surface erosion in peripheral arteries exposes subendothelial matrix and tissue factor, leading to platelet adhesion, thrombin burst, fibrin formation and impaired fibrinolytic balance; distal embolisation and superimposed thrombosis can then convert stable claudication into acute limb ischaemia or accelerate tissue loss [40, 41, 52–54]. Accordingly, biomarker patterns dominated by soluble platelet activation markers, thrombin generation, fibrin turnover, D‐dimer or protein C pathway perturbation should not be viewed as generic inflammatory noise but as candidate indicators of limb‐event propensity and of the biological rationale for intensified antithrombotic therapy [40, 41, 52–54].

Endothelial activation and dysfunction constitute a related but partially distinct axis. Reduced nitric‐oxide bioavailability, oxidative stress, disturbed shear sensing, leukocyte adhesion and barrier dysfunction can precede overt clinical worsening and may be captured by adhesion molecules, thrombomodulin, angiogenic mediators or endothelial cell‐state signatures before major changes in ABI occur [22, 38, 52, 55]. This is important in PAD because endothelial dysfunction links systemic risk factors to both plaque progression and downstream microvascular malperfusion, thereby potentially generating early warning biomarkers even in patients with limited symptoms.

Metabolomics and lipidomics studies provide complementary insight into systemic and muscle‐specific metabolic derangements in PAD. A comprehensive review of PAD metabolomics has underscored consistent perturbations in acylcarnitines, amino acids and organic acids across studies, reflecting impaired mitochondrial *β*‐oxidation, altered glycolytic flux and redox imbalance in ischaemic limb muscle, and emphasised the potential of metabolite panels to improve detection of subclinical disease and to monitor progression [26, 53]. In an Estonian case–control study of male patients with PAD, targeted metabolomics identified a metabolomic signature of arterial stiffness characterised by higher lactate, free carnitine and selected amino acids, accompanied by lower levels of pyruvate, citrate and other tricarboxylic acid cycle intermediates, indicating a shift from oxidative phosphorylation towards anaerobic glycolysis in association with vascular dysfunction [27, 28]. Targeted serum metabolomics in individuals with Type 2 diabetes at risk of critical limb ischaemia has further delineated a CLI‐specific metabolic profile, including short‐chain acylcarnitines and amino acid derivatives that discriminate CLI from less advanced PAD and may assist in early identification of patients at the highest risk of limb loss [29, 30]. Additional metabolomics work in cardiovascular cohorts has linked plasma taurine, carnitine derivatives and sphingolipids, including ceramide species, to atherosclerotic events and lower‐extremity disease, reinforcing the concept that lipidomic perturbations capture both systemic cardiometabolic stress and local vascular injury.

Large‐vessel stiffness adds another interpretive layer. In PAD, arterial stiffness refers to reduced arterial compliance, whereas wave reflections describe the return of pressure waves from distal branch points or stiff segments that augment central systolic load and impair diastolic flow reserve. Stiffer arteries and earlier wave reflections are associated with endothelial injury, chronic inflammation, renal dysfunction and metabolic stress, which helps explain why inflammatory proteins, cystatin C or other renal markers and lactate‐ or carnitine‐rich metabolomic profiles can cluster together in some PAD cohorts [27, 28, 48].

### 3.3. Cross‐Compartment Patterns and Emerging Multiomics Signatures

These vascular, muscular and circulating datasets point to a limited number of recurring multiomics axes in PAD: inflammatory and immune activation, thrombotic and coagulation signalling, dysregulated lipoprotein and lipid metabolism, chronic hypoxia with mitochondrial dysfunction and maladaptive tissue remodelling [56, 57]. Single‐cell and bulk transcriptomic analyses highlight SMC phenotypic modulation, endothelial dysfunction, proinflammatory macrophage polarisation, pericyte expansion and satellite cell exhaustion as central tissue‐level processes, whereas plasma proteomics and metabolomics report concordant signatures in complement/coagulation cascades, acute‐phase proteins, apolipoproteins, acylcarnitines and TCA‐cycle intermediates [55, 58]. Integrative frameworks developed in complex diseases more broadly show that combining genomics with transcriptomic, proteomic and metabolomic profiles in primary tissues can substantially improve the mapping of risk loci to effector genes, pathways, and biomarker candidates, particularly when single‐cell and spatial omics technologies are used to resolve cell type–specific mechanisms [59, 60]. Recent conceptual work in PAD has extended this logic by proposing multiomics pipelines that aggregate arterial wall, muscle and plasma data to identify gene hubs and networks amenable to gene‐ or RNA‐based therapies and to derive composite biomarker panels that track limb ischaemia burden, microvascular remodelling and systemic atherothrombotic risk.

Not all limb ischaemia is biologically equivalent. Predominant large‐vessel stenosis is most likely to generate biomarker patterns related to plaque burden, thrombosis, disturbed shear stress and collateral remodelling; predominant microvascular dysfunction may instead manifest through endothelial, inflammatory and metabolic signatures of impaired capillary recruitment and tissue oxygen extraction; and impaired vasoreactivity can accentuate exercise‐induced ischaemia through defective nitric‐oxide signalling or abnormal neurohumoral responses, leading to exaggerated postexertional metabolic perturbation despite only moderate resting stenosis [8, 20–22, 25–31]. Framing biomarker data against this tripartite model helps explain why patients with apparently similar ABI or angiographic disease may show very different proteomic and metabolomic profiles.

Collateral remodelling and angiogenic competence further modify biomarker expression. Adaptive arteriogenesis and microvascular remodelling recruit endothelial, pericyte, macrophage and matrix‐remodelling programmes involving VEGF‐related, hypoxia‐responsive and extracellular‐matrix pathways [22, 25, 41]. Patients with well‐developed collaterals can therefore maintain tissue perfusion and walking function despite severe proximal stenosis, which may blunt systemic hypoxia or muscle‐injury biomarkers and decouple anatomical severity from circulating signatures. Conversely, poor collateralisation may amplify metabolic stress and wound‐healing failure at a similar degree of macrovessel obstruction.

## 4. Integrative Strategies for Mapping Variant‐to‐Biomarker Pathways in PAD

Integrative strategies for mapping variant‐to‐biomarker pathways in PAD build on the premise that inherited and acquired genomic variation perturbs specific regulatory elements, alters gene and protein networks in vascular and immune cells and generates circulating or imaging readouts that can be measured in patients [61]. Multiomics frameworks developed in ASCVD already demonstrate that combining genomics, epigenomics, transcriptomics, proteomics and metabolomics with clinical data improves causal inference, risk prediction and target prioritisation beyond single‐omic approaches [62, 63]. Within PAD, analogous workflows need to start from robustly replicated susceptibility loci, incorporate disease‐relevant tissue omics, and explicitly evaluate whether candidate molecular traits can serve as clinically useful biomarkers rather than generic correlates of atherosclerotic burden.

A first layer of integration links PAD risk variants to effector genes. In the Million Veteran Program GWAS, 19 loci were identified and subsequently annotated using phenome‐wide association, arterial eQTL, transcriptome‐wide association in tibial artery and plasma protein QTL resources to prioritise candidate genes such as LPA, LPL, LDLR, F5, SORT1 and COL4A1 [64, 65]. These analyses illustrate a general strategy in which fine‐mapped PAD loci are intersected with cis‐regulatory annotations and eQTL data from vascular, hepatic and immune tissues to assign putative effector genes and to distinguish loci acting primarily through lipoprotein metabolism, thrombosis, glycaemic control or vascular matrix remodelling. Integrating single‐cell and spatial transcriptomic datasets from human atherosclerotic plaques into this layer, as recently done for ASCVD, allows variant‐to‐gene assignments to be resolved at the level of specific smooth muscle, endothelial and macrophage states that are directly implicated in lesion progression and instability. A second layer connects effector genes to protein and metabolite biomarkers through quantitative trait locus analyses. Large protein quantitative trait locus (pQTL) studies in community cohorts have mapped tens of thousands of cis and trans pQTLs, integrated them with coronary disease GWAS signals and used Mendelian randomization (MR) to identify circulating proteins with putative causal roles in cardiovascular events. Complementary proteogenomic work in several thousand individuals has shown that serum protein levels share loci with many common diseases, and that these shared loci often overlap known GWAS hits [66, 67]. Recent disease‐focused pQTL analyses extended this approach to 42 clinical phenotypes, including PAD, and demonstrated colocalisation of apolipoprotein(a) variants with PAD, coronary heart disease and major adverse cardiac events, thereby providing direct genetic support for lipoprotein(a)‐related biomarker axes across vascular beds [68, 69]. Population‐specific pQTL work further shows that genetic regulation of inflammatory and cardiometabolic proteins can differ substantially between ancestries, underscoring the importance of diverse cohorts for PAD biomarker discovery [70, 71]. These resources allow PAD risk loci to be systematically queried for effects on plasma proteins and metabolites that lie in lipid, coagulation, inflammatory and hypoxia‐related pathways.

Interpretation of molecular QTLs requires careful terminology. Cis‐QTLs are variants located near the gene or analyte they regulate, commonly within or around the gene locus, whereas trans‐QTLs influence distant genes or proteins, often through upstream signalling or network effects [38, 39, 64–72]. Tissue specificity refers to the observation that the same variant may regulate expression or protein abundance in one tissue, cell type or activation state but not in another, which is especially relevant in PAD where arterial endothelium, smooth muscle, liver, immune cells, and ischemic muscle each capture different biology [22, 38, 55]. Platform effects denote assay‐dependent differences in what is measured, for example aptamer‐ versus antibody‐based proteomics, targeted versus untargeted metabolomics, or bulk versus single‐cell transcriptomics, which can alter dynamic range, isoform specificity, epitope sensitivity and missingness [39, 60, 64–73]. Common pitfalls include attributing a trans signal to the nearest gene, overlooking ancestry‐specific linkage disequilibrium, ignoring cell‐state specificity, mistaking assay artefacts or protein‐altering variants for biological regulation and transferring a QTL discovered in plasma or liver directly to limb muscle or diseased arterial tissue without validation.

Causal variant‐to‐biomarker inference can then be strengthened by MR and colocalisation analyses that treat cis‐pQTLs or cis‐mQTLs as instruments for candidate biomarkers and PAD as the outcome. General cardiovascular pQTL studies have already shown that this combination of pQTL mapping, disease GWAS colocalisation, and MR can identify proteins that both share genetic determinants with disease risk and predict future events [72, 74]. In PAD, a recent MR analysis that integrated seven pQTL datasets with PAD GWAS summary statistics identified four plasma proteins (MMP3, MMP1, CASS4 and ISG15) with evidence for causal association, applied Bayesian colocalisation to confirm shared genetic signals, and used MR Bayesian model averaging to prioritise MMP1 as a leading target [75, 76]. This example illustrates how variant‐anchored MR frameworks can move from susceptibility loci to specific circulating proteins that encode matrix remodelling, cytoskeletal signalling and interferon pathways, and that are directly actionable in drug development.

Because MR is used here as a bridge from variant to biomarker, its core assumptions should be made explicit. The genetic instrument must be associated with the biomarker of interest (relevance), should be independent of major confounders of biomarker‐outcome associations (independence) and should influence PAD only through the biomarker rather than through alternative pathways (exclusion restriction) [71, 72, 74–76]. Horizontal pleiotropy, weak instruments, linkage disequilibrium contamination and sample overlap can violate these assumptions and produce apparently causal results that are actually driven by correlated pathways. Colocalisation is therefore often necessary because it tests whether the biomarker QTL and PAD association are consistent with a shared causal variant; however, it is not sufficient on its own, since a shared variant does not prove directionality, mediation or absence of pleiotropy [71, 72, 74–76]. The most credible variant‐to‐biomarker claims in PAD will generally require concordant evidence from cis‐instrument MR, colocalisation, sensitivity analyses and disease‐relevant functional data.

**Figure 1:**
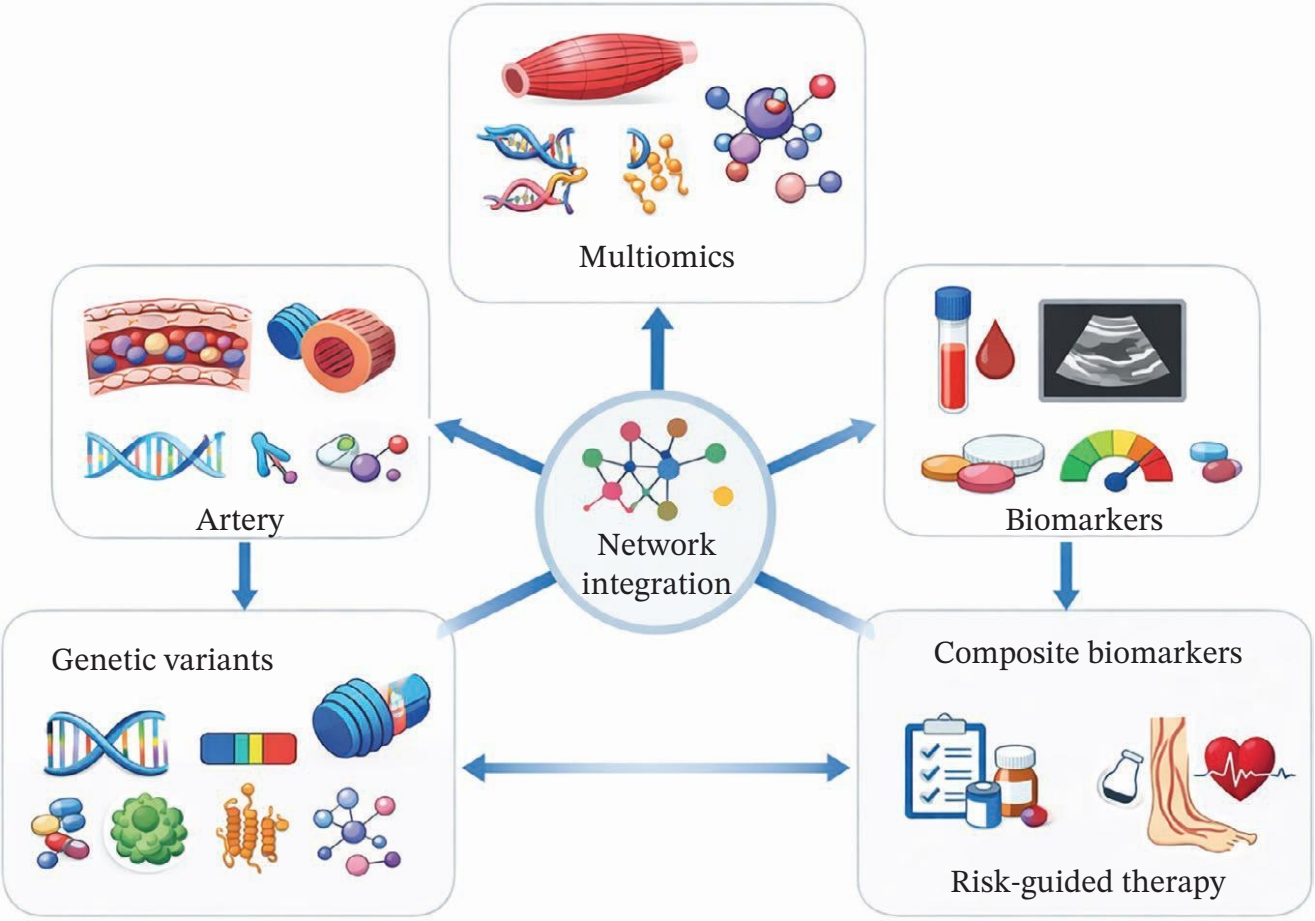
Network integration of variant‐to‐biomarker pathways in peripheral artery disease.

As shown in Figure 1, systems‐level integration across vascular, muscular and circulating compartments provides an additional layer of structure for variant‐to‐biomarker pathways. Network‐based multiomics analyses in ASCVD have used coexpression and coabundance modules to group genes, proteins and metabolites into biologically coherent units, relate these modules to imaging or clinical phenotypes, and test for enrichment of GWAS signals within module members [77]. Applying similar approaches in PAD would involve constructing cross‐compartment networks that include arterial and skeletal muscle transcriptomic signatures, plasma proteomic and metabolomic profiles and genetically informed scores derived from PAD loci [78]. Modules enriched for PAD risk variants and for pQTL‐ or mQTL‐linked proteins would then define molecular endotypes corresponding, for example, to thromboinflammatory activity, dyslipidaemia, or mitochondrial dysfunction and provide candidates for composite biomarker panels that better reflect limb ischaemia biology than single analytes.

In this review, a module refers to a statistically derived set of coexpressed genes, coabundant proteins, covarying metabolites or cross‐omic features that cluster together across samples and are typically summarised by an eigengene, factor score or latent component [60, 73, 77, 78]. An endotype refers to a biologically interpretable patient subgroup defined by one or more replicated modules and linked to distinct mechanisms or treatment responses, not simply any unsupervised cluster. Derivation should be transparent, for example weighted gene coexpression analysis, graphical models, factor analysis or multiview integration, with strict separation of feature selection from model training. Replication should ideally include preservation of module structure, reproducibility of the summary score and consistent association with prespecified phenotypes in an external cohort or on an orthogonal assay platform [60, 73, 77, 78]. To limit overfitting and overinterpretation, PAD multiomics panels should use nested cross‐validation, penalised or sparse models, calibration assessment and external validation, and biological claims should be restrained when a panel improves prediction statistically but lacks mechanistic specificity.

Implementing these integrative strategies in PAD requires attention to several practical issues identified in multiomics work on ASCVD more broadly. Reviews emphasise the need for harmonised preanalytical protocols, careful control of batch effects and adequate sample sizes at each omic layer, as well as inclusion of women and non‐European ancestries to improve generalisability [73]. Access to disease‐relevant tissues remains a limiting factor; for PAD, priority should be given to generating paired multiomics data from femoral or tibial arteries, distal skeletal muscle and plasma in well‐phenotyped cohorts with longitudinal follow‐up for limb and cardiovascular outcomes [9]. Machine‐learning models that combine genetic risk scores, omic module scores and established clinical variables can then be trained to evaluate whether variant‐informed biomarker panels add incremental predictive value over current risk scores, provided that model development follows strict principles of internal cross‐validation and external replication. Under these conditions, variant‐to‐biomarker pathways in PAD can be defined in a way that is mechanistically interpretable, quantitatively robust and directly linked to therapeutic target evaluation.

## 5. Clinical Translation of PAD Biomarkers: Risk Stratification and Therapeutic Guidance

Clinical translation of variant‐informed PAD biomarkers requires that molecular readouts improve risk stratification and therapeutic decision‐making beyond established indices such as the ankle–brachial index. In current practice, guidelines still rely on conventional risk factors and haemodynamic measures, and biomarkers are rarely used in a structured way for treatment selection or monitoring. Bridging this gap demands prospective evidence that genetically anchored or multiomics‐derived biomarkers refine prediction of major adverse cardiovascular events and major adverse limb events.

For clinical translation, the intended role of each biomarker must be explicit. Diagnostic biomarkers aim to detect or confirm PAD or a biologically meaningful subtype; prognostic biomarkers estimate future risk of cardiovascular or limb events; predictive or theragnostic biomarkers identify patients more likely to benefit or be harmed by a given therapy; and pharmacodynamic or monitoring biomarkers quantify biological response over time [8, 79, 80]. In our framework, no current multiomics panel should be viewed as a replacement for ABI or imaging as a stand‐alone diagnostic test. Rather, variant‐informed molecular endotypes are most immediately suited to prognostic enrichment, prediction of response to intensified antithrombotic or lipid‐lowering therapy and pharmacodynamic monitoring after exercise or revascularisation, with diagnostic use most plausible as an adjunct in patients whose symptoms, ABI, and imaging are discordant.

Single circulating biomarkers have provided initial proof‐of‐concept. In a meta‐analysis of 16 studies, higher baseline C‐reactive protein levels were associated with an approximately twofold increase in major cardiovascular events among patients with PAD, independent of traditional risk factors, supporting inflammation as a prognostic axis [81]. Other studies have shown that homocysteine, albuminuria and the neutrophil‐to‐lymphocyte ratio each convey additional information on all‐cause and cardiovascular mortality, and that combining these markers into a continuous multimarker score improves risk discrimination compared with clinical variables alone [82]. These findings indicate that composite biomarker profiles reflecting thromboinflammation, endothelial dysfunction and renal microvascular injury identify PAD subgroups at high systemic risk.

Multibiomarker and multiomics panels extend this concept by capturing broader axes of arterial, muscular and metabolic pathology. In a population‐based analysis, proteomic signatures comprising complement, coagulation, apolipoprotein and growth‐factor pathways correlated with global atherosclerotic burden and improved cardiovascular risk prediction on top of conventional scores [83]. Metabolomic profiling across atherosclerotic phenotypes has highlighted acylcarnitines, amino acids and organic acids as markers of mitochondrial dysfunction and altered energy metabolism. Although PAD‐specific proteomic and metabolomic studies remain relatively small, several have reported that multianalyte panels predict incident PAD or adverse events more accurately than individual metabolites. As shown in Table 3, candidate clinical applications for such panels span baseline risk stratification, assessment of residual risk under standard therapy and monitoring of limb ischaemia burden and treatment response.

**Table 3:** Biomarker categories in peripheral artery disease and their representative domains, primary clinical applications and translational considerations.

Biomarker category and context of use	Biomarker domain	Example markers or scores	Potential clinical decision
Prognostic risk stratification for cardiovascular and limb events	Inflammation and thromboinflammation	High‐sensitivity CRP, IL‐6, neutrophil‐to‐lymphocyte ratio and composite inflammatory scores	Identify patients requiring intensified risk‐factor control, closer follow‐up or escalation of antithrombotic therapy
Diagnostic adjunct for limb‐specific ischaemic burden and tissue injury	Metabolic and muscle‐related signatures	Panels of acylcarnitines, amino acids and organic acids; muscle injury proteins	Support diagnostic clarification when ABI and symptoms are discordant, and guide timing and intensity of supervised exercise therapy, imaging and consideration of revascularisation
Predictive/theragnostic assessment of residual risk under guideline‐directed medical therapy	Lipoprotein and coagulation axes	Lipoprotein(a), remnant lipoprotein measures, coagulation factor activities, thromboinflammatory protein modules	Select patients for additional lipid‐lowering, emerging lipoprotein(a)–targeted agents or intensified antithrombotic regimens
Predictive/theragnostic prediction of treatment response and adverse events	Genetic and polygenic scores; pharmacodynamic biomarker changes	PAD polygenic risk scores, variant‐linked proteomic modules, on‐treatment biomarker trajectories	Tailor primary prevention and antithrombotic strategies, identify individuals with high ischaemic benefit or excess bleeding risk
Pharmacodynamic/monitoring of disease progression and therapeutic effect	Cross‐compartment multiomics panels and simplified composite scores	Integrated protein and metabolite scores aligned with ankle–brachial index, walking distance and limb perfusion	Track progression, evaluate response to revascularisation or exercise, and trigger treatment intensification or de‐escalation

ABI alone is particularly limited in diabetes. Two patients can share the same resting ABI but have very different infrapopliteal disease burden, medial arterial calcification, microvascular dysfunction, neuropathy, infection burden and wound‐healing reserve; thus, one may remain stable while the other progresses to ulceration, chronic limb‐threatening ischaemia or amputation [1, 8, 29, 30, 33]. Biomarkers reflecting endothelial injury, inflammation, glycaemic stress, coagulation and muscle metabolism may therefore be especially useful in diabetes to contextualise an ABI that is either deceptively normal or insufficiently granular for limb‐risk estimation.

Genetic and polygenic biomarkers offer a complementary dimension focused on inherited susceptibility and lifetime risk. Polygenic risk scores derived from large PAD GWAS and tested in cardiometabolic trial cohorts have been associated with prevalent PAD and with major adverse limb events independently of established risk factors, but the gain in discrimination over clinical models has so far been modest [84]. These observations suggest that polygenic scores may be most useful for early‐life risk communication, selection of individuals for intensive primary prevention and anchoring downstream omics‐based biomarker discovery rather than as stand‐alone tests in advanced disease. Integration of polygenic scores with plasma proteomic modules and tissue transcriptomic signatures linked to specific PAD loci could delineate molecular endotypes in which therapeutic strategies are more likely to be effective.

The clearest evidence for biomarker‐guided therapeutic strategies in PAD currently relates to antithrombotic intensification. A meta‐analysis combining PAD cohorts from the COMPASS and VOYAGER trials showed that dual‐pathway inhibition with low‐dose rivaroxaban plus aspirin, compared with aspirin alone, reduced a composite of cardiovascular death, myocardial infarction, ischaemic stroke, acute limb ischaemia and major vascular amputation at the cost of an increased risk of major but not fatal or critical‐organ bleeding [54]. In clinical practice, inflammatory and thrombotic biomarker profiles could be used to enrich for patients with high absolute risk and low bleeding propensity, in whom the net clinical benefit of intensified antithrombotic therapy is maximised. Similar principles may apply to emerging interventions targeting lipoprotein(a), residual triglyceride‐rich lipoprotein remnant burden or specific inflammatory pathways, for which variant‐to‐biomarker mapping can help identify responsive subgroups.

Therapy can also reshape the biomarker landscape, which is clinically useful but analytically hazardous. Statins and intensive lipid lowering can reduce apoB‐related proteins, inflammatory mediators and some plaque‐associated proteomic signatures; antithrombotic regimens can alter thrombin generation, fibrin turnover, platelet‐activation markers and endothelial‐surface shedding; glucose‐lowering therapies may shift acylcarnitines, ketone‐related metabolites and inflammatory tone; successful revascularisation can normalise ischaemia‐driven metabolite gradients and reduce muscle‐injury signals; and supervised exercise can induce repeated acute metabolic excursions while improving mitochondrial efficiency and endothelial function over time [20, 21, 26, 30, 31, 52–54]. For this reason, proteomic or metabolomic panels should be interpreted with careful adjustment for treatment exposure and, when possible, distinguished as baseline prognostic markers versus pharmacodynamic monitoring markers.

Translating PAD biomarkers into routine care will require evaluation of analytical validity, clinical validity and clinical utility. Analytical considerations include assay standardisation, stability across laboratories and platforms and the feasibility of measuring multianalyte panels in a time frame compatible with outpatient decision‐making. Clinical validity must be established in diverse PAD populations with clear definitions of cardiovascular and limb end points, using metrics such as discrimination, calibration and reclassification that reflect both systemic and limb‐specific risk. Clinical utility depends on randomized or pragmatic trials showing that biomarker‐enriched treatment strategies alter management and improve outcomes, for example by guiding initiation of dual‐pathway inhibition, advanced lipid‐lowering or early revascularisation.

Interpretation of inflammatory signatures also depends on how exposures are measured. Objective measures such as nicotine metabolites, HbA1c and other glycaemic indices, accelerometry‐derived physical activity, renal function and pharmacy fill data often outperform simple self‐report because they reduce recall error and social‐desirability bias and better align molecular readouts with true biological exposure [6, 9, 20, 21, 42]. In PAD cohorts, this is especially relevant because smoking intensity, diabetes control, sedentary behaviour and medication adherence can all shift inflammatory and thrombotic profiles and may otherwise be mistaken for disease‐specific endotypes.

The design of future variant‐to‐biomarker frameworks for PAD should define their intended context of use and align that context with feasible assays and decision algorithms. Multiomics studies in ASCVD demonstrate that integrating genomics, transcriptomics, proteomics and metabolomics with clinical phenotyping can identify molecular modules that both capture disease activity and point to druggable pathways [83, 85]. For PAD, priority areas include the development of parsimonious biomarker panels that approximate complex omics signatures, validation of these panels across ancestral groups and disease stages and incorporation into decision‐support tools embedded in electronic health records.

Clinically, biomarker interpretation should be anchored to outcomes that matter to patients. For PAD, these include walking distance and speed, exertional leg symptoms, health‐related quality of life, wound healing, freedom from major amputation and amputation‐free survival rather than only composite cardiovascular events [5, 9, 11, 24, 86]. A limb‐metabolic panel that correlates with a 6‐min walk decline or calf muscle pathology may have little relevance for antithrombotic choice, whereas a thromboinflammatory panel may inform acute limb risk but not day‐to‐day walking limitation. Explicit linkage of each biomarker domain to functional, tissue‐healing and survival endpoints is therefore essential for clinical translation.

## 6. Conclusion and Perspectives

PAD exemplifies the challenge and promise of variant‐to‐biomarker research in complex cardiovascular disorders. Current evidence indicates that PAD risk is shaped by a polygenic background of common variants of modest effect, superimposed on conventional cardiometabolic risk factors and environmental exposures. Multiomics studies across arterial wall, limb muscle and circulation converge on a limited set of recurrent biological themes, including dysregulated lipoprotein and lipid metabolism, thromboinflammation, chronic hypoxia with mitochondrial dysfunction and maladaptive tissue remodelling [87, 88]. Bringing these elements together in explicit variant‐to‐biomarker pathways provides a coherent framework for understanding how inherited susceptibility and molecular network perturbations translate into measurable clinical phenotypes in the lower extremities.

**Figure 2:**
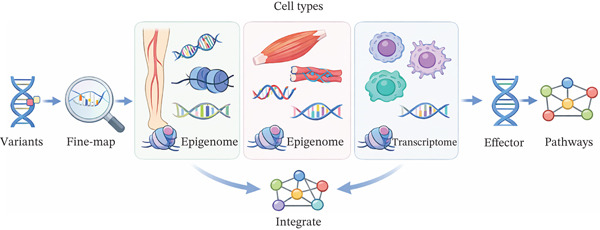
Cell type–resolved multiomics integration to map PAD variants to effector genes and pathways.

As shown in Figure 2, at the mechanistic level, integrating fine‐mapped PAD loci with cell type–resolved epigenomic and transcriptomic data from femoral and tibial arteries, skeletal muscle and immune cells can refine the assignment of effector genes and pathways. When these variant‐to‐gene links are combined with protein and metabolite quantitative trait loci, and with network‐based analyses that span vascular, muscular and circulating compartments, they begin to define molecular endotypes of PAD [89, 90]. Such endotypes may correspond to predominant thrombotic, inflammatory, dyslipidaemic or hypoxia–metabolic axes and naturally suggest biomarker candidates at each layer, from cell state signatures and pathway modules to composite plasma or imaging readouts that quantify limb ischaemia burden and systemic atherothrombotic risk.

From a translational perspective, the key question is whether variant‐informed biomarker panels can improve risk stratification, therapeutic selection and monitoring beyond established indices such as the ankle–brachial index and conventional risk scores [79, 80]. Early work on inflammatory, thrombotic, lipid and metabolic markers, together with polygenic scores derived from PAD GWAS, supports the concept that multianalyte and multiomics panels can modestly enhance prediction of major cardiovascular and limb events and reveal heterogeneity in treatment benefit. However, the incremental gain in discrimination has generally been small, context‐specific and sensitive to ancestry, disease definition and assay platform [91, 92]. These observations emphasise that the clinical value of PAD biomarkers will depend not only on their statistical performance, but also on clearly defined contexts of use, parsimonious panel design and rigorous evaluation of net benefit in decision‐relevant settings.

Several methodological, logistical and ethical challenges must be addressed before variant‐to‐biomarker pathways in PAD can be routinely deployed in clinical practice. Access to well‐characterised primary tissues remains limited, particularly for distal arteries and limb muscle across the spectrum from asymptomatic disease to chronic limb‐threatening ischaemia. Multiomics studies have often been restricted to modest sample sizes, single ancestries and cross‐sectional designs, which constrains power for fine‐mapping, causal inference and external validation. Standardisation of preanalytical handling, data generation and computational pipelines is still incomplete, hindering robust meta‐analysis and reproducibility. In parallel, implementation of complex biomarker assays in everyday care will require attention to cost, turnaround time, regulatory approval, data governance and equitable access across health systems.

Future progress will depend on coordinated efforts to generate larger, ancestrally diverse and deeply phenotyped PAD cohorts with harmonised clinical end points and longitudinal follow‐up. Within such cohorts, systematic acquisition of genomics, epigenomics, single‐cell and spatial transcriptomics, proteomics and metabolomics from vascular and muscular tissues, combined with matched plasma profiling, will be essential to delineate cross‐compartment networks anchored in PAD risk loci. High‐throughput functional genomics, including CRISPR‐based perturbation screens in disease‐relevant cell types, can help resolve causal variants and effector genes within risk loci, whereas interpretable machine learning models that integrate sequence, chromatin and network features may prioritise regulatory variants and biomarker candidates for experimental validation. Conceptually, this trajectory parallels work in other complex traits that seek to move from statistical association to functional interpretation of noncoding variants and clinically useful biomarkers.

In outlook, variant‐to‐biomarker research in PAD is positioned to evolve from proof‐of‐concept studies towards clinically actionable tools that support precision prevention and treatment of limb and cardiovascular events. Over the coming years, priority should be given to developing simplified biomarker panels that approximate complex multiomics signatures, validating these panels across ancestries and disease stages, and embedding them in decision‐support algorithms integrated with electronic health records. Prospective, biomarker‐enriched trials will be needed to test whether such strategies can identify patients who derive disproportionate benefit from intensified antithrombotic, lipid‐lowering, anti‐inflammatory or revascularisation therapies, without unacceptable increases in harm. If these conditions are met, variant‐informed biomarker pathways have the potential to transform PAD care from a late‐diagnosed and undertreated condition into one in which inherited susceptibility and molecular disease activity are quantified and acted upon in a timely manner.

Near‐term adoption is therefore most plausible where biomarkers are tied to explicit contexts of use, namely prognostic enrichment, predictive selection of intensified therapy and pharmacodynamic monitoring, and to patient‐centred outcomes such as walking function, wound healing and amputation‐free survival.

## Author Contributions

Wanting Wang and Gang Zhao contributed to the literature search, data curation and initial drafting of the manuscript. Changxin Yang and Siyao Chang conceived the review topic, designed the overall structure, supervised the writing process and critically revised the manuscript for important intellectual content.

## Funding

No funding was received for this manuscript.

## Disclosure

All authors read and approved the final version of the manuscript.

## Conflicts of Interest

The authors declare no conflicts of interest.

## Data Availability

Data sharing is not applicable to this article as no datasets were generated or analysed during the current study.
